# Acute knockdown of the insulin receptor or its substrates Irs1 and 2 in 3T3-L1 adipocytes suppresses adiponectin production

**DOI:** 10.1038/srep21105

**Published:** 2016-02-18

**Authors:** Matthijs P. Groeneveld, Gemma V. Brierley, Nuno M. Rocha, Kenneth Siddle, Robert K. Semple

**Affiliations:** 1 The University of Cambridge Metabolic Research Laboratories, Wellcome Trust-MRC Institute of Metabolic Science, Cambridge, UK; 2 The National Institute for Health Research Cambridge Biomedical Research Centre, Cambridge, UK

## Abstract

Loss of function of the insulin receptor (*INSR*) in humans produces severe insulin resistance. Unlike “common” insulin resistance, this is associated with elevated plasma levels of the insulin-sensitising, adipose-derived protein adiponectin. The underlying mechanism for this paradox is unclear, and it is at odds with the acute stimulation of adiponectin secretion reported on insulin treatment of cultured adipocytes. Given recent evidence for ligand-independent actions of the INSR, we used a lentiviral system to knock down *Insr* or its substrates *Irs1* and *Irs2* conditionally in 3T3-L1 murine preadipocytes/adipocytes to assess whether acute loss of their expression has different consequences to withdrawal of insulin. Efficient knockdown of either *Insr* or *Irs1/2* was achieved by conditional shRNA expression, severely attenuating insulin-stimulated AKT phosphorylation and glucose uptake. Dual knockdown of *Irs1* and *Irs2* but not *Insr* in preadipocytes impaired differentiation to adipocytes. Acute knockdown of *Insr* or both *Irs1* and *Irs2* in adipocytes increased *Adipoq* mRNA expression but reduced adiponectin secretion, assessed by immunoassay. Knockdown sustained for 14 days also reduced immunoassay-detected adiponectin secretion, and moreover induced delipidation of the cells. These findings argue against a distinct effect of Insr deficiency to promote adiponectin secretion as the explanation for paradoxical insulin receptoropathy-related hyperadiponectinaemia.

Insulin drives macronutrient storage and tissue growth by inducing trans-autophosphorylation of its receptor, which is a dimeric transmembrane receptor tyrosine kinase (RTK). This results in phosphorylation of insulin receptor substrates (IRSs) 1 and 2 and activation of a widely ramifying signalling network including, but not limited to, the phosphatidylinositol-3-kinase/AKT/mTOR and RAS/MEK/ERK pathways^1^.

Insulin resistance is commonly associated with type 2 diabetes mellitus, fatty liver, dyslipidaemia and ovulatory dysfunction^2^. However loss of insulin receptor (INSR) function produces a distinctive insulin resistance subphenotype, with severely impaired responsiveness of blood glucose levels to insulin and subfertility but neither fatty liver disease nor dyslipidaemia^3^. Moreover, while plasma levels of the abundant adipose-derived protein adiponectin are lowered in prevalent insulin resistance^4^ they are preserved or increased, sometimes extremely, in INSR dysfunction^5^,^6^. Hyperadiponectinaemia in mice with adipose- specific *Insr* knockout^7^ implicates increased adiponectin production rather than reduced clearance in this. Insulin stimulates adiponectin secretion from cultured adipocytes (e.g^8^), however, at odds with the *in vivo* observations. These findings could be reconciled if the INSR has ligand-independent functions relevant to adiponectin production. Evidence for ligand-independent INSR functions has recently emerged with the finding that its knockout confers resistance to apoptosis upon murine brown preadipocytes if *Igf1r* is concomitantly deleted^9^.

*INSR* is commonly co-expressed with *IGF1R*, which activates a nearly identical signalling pathway, yet their biological effects are distinct. This is likely to be accounted for in part by tissue expression profiles of the receptors^1^. The role of adipocyte IGF1R homodimer is minor or negligible compared to that of the INSR^10^ and so in adipocytes ligand-independent effects of the INSR may be physiologically relevant. To assess whether loss of non ligand-dependent actions of the INSR accounts for the hyperadiponectinaemia of insulin receptoropathy, we conditionally knocked down *Insr* or *Irs1* and *Irs2* (*Irs1/2*) in murine 3T3-L1 adipocytes.

## Results and Discussion

**Figure 1:**
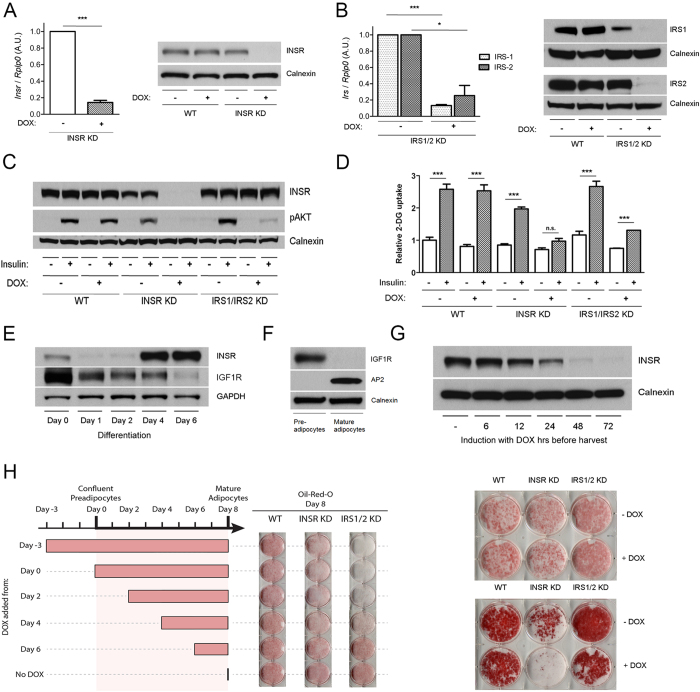
Conditional knockdown of *Insr* or *Irs1/Irs2* in 3T3-L1 adipocytes. 3T3-L1 adipocytes harbouring DOX-inducible miR-shRNAs targeting the *Insr* (INSR KD) or *Irs1* and 2 (IRS1/2 KD) were exposed to DOX for 72 hours from day 7 of differentiation. (**A**) *Insr* mRNA and protein levels in INSR KD cells compared to controls (**B**) *Irs1* and *Irs2 *mRNA and protein levels in IRS1/2 KD cells. (**C**) AKT Ser473 phosphorylation after serum starvation and exposure to 10nmol/l insulin for 5 minutes. (**D**) 2-deoxyglucose uptake after exposure to 50nmol/l insulin. (**E**) Western blot analysis of Insr and Igf1r expression in differentiating wild-type (WT) 3T3-L1 pre-adipocytes. Days post initiation of differentiation are shown. (**F**) Expression of Igf1r and aP2 in isolated, lipid-laden WT 3T3-L1 adipocytes after 6 days of differentiation. (**G**) Time-course of Insr protein expression in differentiated 3T3-L1 cells in response to DOX. (**H**) Oil-Red-O staining of WT, INSR and IRS1/2 KD cells differentiated for 8 days. DOX was added at the timepoints indicated in the schematic. (**I**) Oil-Red-O staining of 3T3-L1 WT, INSR KD and IRS1/2 KD cells ± doxycycline for 72 hours. Images are representative of 3 independent experiments. (**J**) Oil-Red-O staining of 3T3-L1 WT, INSR KD and IRS1/2 KD cells ± doxycycline for 14 days from day 7 of differentiation. Images are representative of 3 independent experiments. Error bars represent mean ± S.E.M. from 3 independent experiments. Paired two-tailed Student’s t test; *denotes p < 0.05, ***p < 0.001 and non-significant (n.s.) indicates p > 0.05.

**Figure 2:**
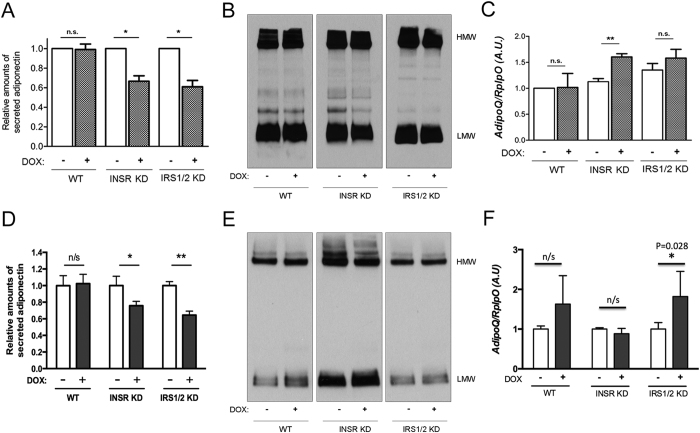
Effect on adipocyte adiponectin synthesis and secretion of *Insr* or *Irs1/Irs2* knockdown. 3T3-L1 preadipocytes were differentiated for 7 days prior to induction of shRNA expression using DOX for 72 hours (**A–C**) or 14 days (**D–F**). (**A**) Adiponectin concentrations determined by DELFIA assay in medium conditioned for 24 hours. (**B**) Adiponectin in 24 hour conditioned medium determined by non-denaturing, non-reducing polyacrylamide gel electrophoresis and immunoblotting. (**C**) Cellular *AdipoQ* expression determined by quantitative real time PCR mRNA levels and normalised to expression of *Rplp0*. (**D**–**F**) show the same analyses after 14 days of exposure to DOX. Western blots are representative of five independent experiments. Error bars represent mean ± standard error of the mean from at least 3 independent experiments. Paired two-tailed Student’s t test was used to test significance; *p < 0.05; **p < 0.01; n.s. (non-significant) indicates p > 0.05.

3T3-L1 preadipocyte lines were generated allowing knockdown of *Insr* or *Irs1/2* by doxycycline-dependent expression of shRNA. Clonal cell lines were screened for knockdown efficiency, and subsequent studies undertaken using the most efficient lines. After differentiation to adipocytes highly efficient knockdown of *Insr* or *Irs1/2 *mRNA and protein was induced by 72 hours of doxycycline treatment (Fig. 1A,B). Knockdown after differentiation did not affect cellular lipid content (Fig. 1I) but severely attenuated insulin-induced Akt phosphorylation (Fig. 1C) and 2-deoxyglucose uptake (Fig. 1D). Insulin-dependent glucose uptake depends upon Akt, and half maximal uptake requires an Akt phosphorylation level of only 5–22% of its maximum^11^. Thus severe blunting of this response confirms potent *Insr* and *Irs1/2* knockdown.

One challenge when using shRNA to study gene function in adipocytes is that some genes of interest are also involved in preadipocyte differentiation, and their stable knockdown precludes efficient adipocyte generation. Early studies using genetically engineered 3T3 cells suggested that Insr function is required for adipogenesis, although prolonged passage of cells may have reduced the differentiative capacity of the cells^12^. Recent studies using cre-mediated gene deletion in murine primary brown preadipocytes have instead suggested that Insr and Igf1r play redundant roles in early adipogenesis, and that the Insr is thus dispensable for the process^13^.

Our cellular model of inducible *Insr* knockdown enabled us to re-address this question in 3T3-L1 cells. In keeping with previous reports (e.g.^14^) Insr expression was up-regulated during differentiation (Fig. 1E), while Igf1r expression decreased (Fig. 1E). Moreover, expression of Igf1r was not detectable after fractionation of lipid-rich cells to remove residual undifferentiated cells (Fig. 1F). On doxycycline treatment of preadipocytes Insr protein was reduced after 12 hours, near complete knockdown being achieved at 72 hours (Fig. 1G). Knockdown induced between day −3 and day 6 of differentiation only modestly impaired triglyceride accumulation (Fig. 1H), while Irs1/2 knockdown impaired lipidation more severely (Fig. 1H). Insr knockdown for 14 days after adipocyte differentiation led to striking delipidation of the cells (Fig. 1J).

These findings suggest that in the 3T3-L1 adipocyte cell line, as in murine brown primary preadipocytes^9^, Insr plays a predominant role only in the later phase of adipogenesis, when it is highly expressed relative to Igf1r. Indeed, although *Insr* knockout mice die before day 3 of postnatal life with reduced fat cell mass, adipocytes are detectable, indicating that the role of the Insr in adipogenesis *in vivo*, too, is not obligate^12^. The more deleterious effect of *Irs1/2* knockdown is consistent with previous findings^15^, and may be accounted for by their involvement in both Insulin and IGF1 signalling.

Our study was primarily motivated by the unexplained discordance in patients with loss of Insr function between elevated adiponectin and severe insulin resistance^5^,^6^. We thus sought to use our model of conditional Insr deficiency to test whether non ligand-dependent actions of the Insr may be important for regulation of adiponectin secretion. After inducing knockdown in differentiated 3T3-L1 adipocytes, secreted adiponectin was measured over 24 hours using a DELFIA assay and immunoblotting. Both *Insr* and *Irs1/2* knockdown reduced adiponectin secretion assessed by immunoassay (Fig. 2A), although the effect was not apparent in non-denaturing, non-reducing immunoblots, where the complex higher order structure of adiponectin renders interpretation more complex (Fig. 2B). *AdipoQ* mRNA, encoding adiponectin, was increased in adipocytes by *Insr* knockdown, however the difference between *Irs1/2* knockdown cells and doxycycline-free controls was not significant (Fig. 2C).

Some previous data suggest that the acute increase in adiponectin secretion seen on insulin treatment is transient and induced by altered endoplasmic reticulum redox tone^8^. It thus remains possible that increased *AdipoQ* mRNA is more relevant to the *in vivo* setting, in keeping with reports that in humans low plasma adiponectin corresponds to low adipose *ADIPOQ* mRNA^16^,^17^. Knockdown of either *Insr* or *Irs1/2* for 2 weeks in adipocytes once again decreased adiponectin secretion as assessed by immunoassay (Fig. 2D), with no difference discerned by immunoblotting (Fig. 2E). *AdipoQ* mRNA expression showed no significant response to *Insr* knockdown, but was modestly increased by *Irs1/2* knockdown (Fig. 2F). These findings argue against the hypothesis that insulin has divergent acute and long-term effects on adiponectin secretion.

Our findings do not support the notion that the hyperadiponectinaemia of insulin receptoropathy is explained by consequences of INSR deficiency on adipocyte-autonomous adiponectin expression or secretion, however are in keeping with a preponderance of prior studies assessing the consequences of insulin stimulation of adipocytes. The human biochemical paradox thus remains unexplained. Culture conditions used may not adequately mimic the *in vivo* cellular milieu, or the adipocytes studied may not represent the adipose depot driving the *in vivo* phenomenon^22^. Alternatively, loss of INSR function may affect adiponectin levels indirectly through alteration of adipocyte turnover. Further insights may require study of different adipose depots from patients with loss of INSR function.

## Methods

### Lentivirus production and Infection

4–6 murine miR-shRNAs from Open Biosystems were screened per target and the most potent miR-shRNAs identified were cloned into either pSLIK-NEO or pTRIPZ-PURO (Open Biosystems) lentiviral expression vectors, as previously described^18^, and targeted the sequences: CTGGGACTGGAGCAAACACAA (*Insr*), GGATCCCATATCAGTTTCTAA (*Insr*), TTGGGTG- GAGAGAGTATTAAA (*Irs1*) and ACTCGGACAGCTTCTTCTTCA (*Irs2*). Virus was packaged by transfecting the lentivector expression vectors along with third-generation lentivirus packaging and pseudotyping plasmids (pMDLg/pRRE, pRSVREV and pVSV-G) into HEK293T cells using the calcium phosphate transfection method (Clontech). 3T3-L1 preadipocytes were infected with pTRIPZ virus at low MOI to ensure that most transduced cells contained single integrants. After puromycin selection cells were infected with pSLIK, also at low MOI, and selected with both G418 and puromycin.

### Cell culture

3T3-L1 preadipocyte maintainance and differentiation were performed as previously described^19^. Experiments were undertaken at day 7 of differentiation unless otherwise indicated.

### Lipid droplet accumulation

Lipid droplet accumulation in mature adipocytes was visualized by Oil-red-O staining as previously described^15^.

### Insulin stimulation studies and glucose uptake assays

For insulin stimulation studies, cells were washed twice with warm PBS and serum starved in serum-free DMEM medium containing 0.5% bovine serum albumin 16 hours before insulin stimulation. Deoxyglucose uptake was assessed on day 10 of differentiation in 12-well plates essentially as previously described^20^.

### Measurement of secreted adiponectin

For determination of secreted adiponectin, confluent adipocyte cultures were washed with warm PBS before adding 2.0 ml DMEM containing 10% (v/v) FBS. Medium was collected after 24 hours and used for auto-dissociation-enhanced lanthanide fluorescence immunoassay (DELFIA) of adiponectin as described previously^21^.

### Western blot

Cells were washed with cold PBS, snap frozen in liquid nitrogen, thawed and scraped in modified RIPA buffer (substitution of 1% (v/v) Triton-X 100 instead of SDS) containing Complete Mini protease inhibitor cocktail (Roche). Protein concentrations were determined with the Bradford protein assay (Bio-Rad). Where indicated, mature adipocytes were isolated from undifferentiated cells employed trypsinisation of cells after differentiation before suspension in 25ml DMEM and centrifugation at 360 ×g for 9 minutes. Floating adipocytes were transferred to a new tube and centrifuged at 4,255 ×g for 7 minutes before lysing of the pellet. After SDS–polyacrylamide gel electrophoresis and transfer to a polyvinylidene difluoride membrane using the iBlot dry blotting system (Invitrogen) and immunoblotted with the appropriate antibodies, proteins were visualized using enhanced chemoluminescence (GE Healthcare or Millipore). When necessary blots were stripped and reblotted using “Re-blot Plus” (Millipore). Antibodies (with catalogue number) used for Western blot were: INSR (sc-711), IGF1R (sc-713) were purchased from Santa Cruz Biotechnologies. Phospho-Ser473-AKT (9271), IRS1 (2382) and IRS2 (3089) were purchased from Cell Signalling. Adiponectin (MAB3608) was purchased from Milipore. Calnexin (ab75801), GAPDH (ab8245), AP2 (ab66682) and secondary antibodies: horseradish peroxidase (HRP)–conjugated anti-rabbit and anti-mouse immunoglobulin G (IgG) were purchased from (Abcam).

### RNA isolation and Real-time PCR analysis

Total RNA was extracted using an RNeasy mini kit including on-column DNAseI digestion (Qiagen). RNA was reverse- transcribed using M-MLV Reverse Transcriptase (Promega) according to the manufacturer’s instructions. Taqman real time PCR was performed starting with 10 ng of cDNA, sense and antisense oligonucleotides (333 nM) and a 5′-[6FAM], 3′-[TAMRA]- labeled fluorogenic probe (167 nM) (Sigma-Aldrich) using PCR Taqman Mastermix (Applied Biosystems) in a final volume of 12 μl. Fluorescence was monitored and analysed in an ABI Prism 7900 HT sequence detection system (Applied Biosystems). Quantification utilised the relative standard curve method with expression of the gene of interest normalized to that of the reference gene *Rplp0*. Primer and probe sequences (5′ - 3′) were as follows: (*Insr* sense primer) CAATGGGACCACTGTATGCATTCT, (*Insr* antisense primer) GTCCGGCACGTACACGAAGA, (*Insr* probe) TGAGTACCTCAGTGCCAGTGATGTGTTTCC, (*AdipoQ* sense primer) CAGTGGATCTGACGACACCAA, (*AdipoQ* antisense primer) TGGGCAGGATTAAGAG- GAACA, (*AdipoQ* probe) GGGCTCAGGATGCTACTGTTGCAAGC, (RPLP0 sense primer) GGACCCGAGAAGACCTCCTT, (*Rplp0* antisense primer) TCAATGGTGCCTCTGGAGATT, (*Rplp0* probe) CCAGGCTTTGGGCATCACCACG, (*Irs1* Taqman assay, Applied Biosystems) Mm01278327-m1, (*Irs2* Taqman assay, Applied Biosystems) Mm03038438-m1.

### Statistical Analysis

Data are presented as mean ± standard error of the mean (SEM) from three independent experiments. Paired two-tailed Student’s t-test were used to determine statistical significance and were calculated using GraphPad Prism.

## Additional Information

**How to cite this article**: Groeneveld, M. P. *et al.* Acute knockdown of the insulin receptor or its substrates Irs1 and 2 in 3T3-L1 adipocytes suppresses adiponectin production. *Sci. Rep.* **6**, 21105; doi: 10.1038/srep21105 (2016).
